# Impaired Eating and Swallowing Function in Older Adults in the Community: The Kurihara Project

**DOI:** 10.3390/ijerph16204040

**Published:** 2019-10-22

**Authors:** Kyoko Takahashi, Katsuaki Amemiya, Masahiro Nakatsuka, Kei Nakamura, Mari Kasai, Kenichi Meguro

**Affiliations:** 1 Geriatric Behavioral Neurology Project, Tohoku University New Industry Creation Hatchery Center (NICHe), Sendai 980-8575, Japan; rdjjs639@ybb.ne.jp (K.T.); amemy_junten@yahoo.co.jp (K.A.); nakatsuka@med.tohoku.ac.jp (M.N.); knkmr@med.tohoku.ac.jp (K.N.); sato-mari@umin.ac.jp (M.K.); 2 Division of Nuclear Medicine, Tohoku University CYRIC, Sendai 980-8575, Japan

**Keywords:** eating, swallowing, older adults, dementia, community

## Abstract

*Introduction*: Older adults with dementia often develop aspiration pneumonia as a complication due to deterioration of swallowing function. Herein, we report our findings of eating and swallowing-related functions in elderly local residents. *Methods*: The subjects were 229 elderly residents in Kurihara City, including 97 healthy (Clinical Dementia Rating (CDR): 0), 108 with mild cognitive impairment (MCI) (CDR: 0.5), and 24 with dementia (CDR: 1 or higher: CDR 1+). We analyzed the relationships between the findings, eating, and swallowing, based on the database of the Kurihara Project performed from 2008 to 2010. *Results*: In the CDR 0.5 group, some deterioration in oral condition, oral function and swallowing function was confirmed. In the CDR 0.5 group, tooth staining, decrease in oral diadochokinesis (oral motion velocity), increased number of points below the cut-off value in a repetitive saliva swallowing test and the questionnaire, and prolonged water swallowing time were confirmed. In the CDR 1+ group, bad breath, elimination of the pharyngeal reflex, increase in disturbed soft palate elevation, and prolonged jelly swallowing time were confirmed. *Conclusions*: Deterioration of swallowing function was confirmed, even in subjects with mild dementia, in addition to development of problems related to food intake.

## 1. Introduction

Eating and swallowing is a process that involves the recognition of food, conveying of food to the mouth, biting, and swallowing. Food intake is divided into the preceding stage and the preparation stage, while swallowing is divided into the oral phase, the pharyngeal phase, and the esophageal period.

Older adults with dementia often develop aspiration pneumonia as a complication due to deterioration of swallowing function. Aspiration pneumonia is one of the well-known critical and fatal diseases, causing a decreased quality-adjusted lifespan [1]. Most pneumonia arises following the aspiration of microorganisms from the oral cavity or nasopharynx. The aspiration pneumonia is pneumonitis resulting from the altered clearance defenses noted above. A true aspiration pneumonia, by convention, usually refers to an infection caused by less virulent bacteria, primarily anaerobes, which are common constituents of the normal flora in a susceptible host prone to aspiration.

In local communities, oral exercises, etc. are performed to maintain swallowing function in nursing care prevention projects [2]. In hospitals, speech therapists (ST) and nurses provide swallowing rehabilitation for the prevention of aspiration pneumonia and continuous oral food intake. The severity of dementia and swallowing function in patients with cerebral injury at various sites have not yet been clarified sufficiently.

For dementia and pre-dementia stage or mild cognitive impairment (MCI), Delwel et al. [3] reported that swallowing ability and chewing ability were perceived as good for most participants with MCI and dementia, although there were several reports that swallowing dysfunction would occur in 30–60% of older adults [4,5]. Using the Standardized Swallowing Assessment (SSA), Yang et al. [6] reported that men with non-amnestic MCI were more likely to have dysphagia than those without non-amnestic MCI, whereas this difference was not found in men with amnestic MCI and women. There were several reports showing that dysphagia was not observed in many patients with early-stage dementia, especially Alzheimer disease (AD) [7,8]; however, Horner et al. [9] reported that AD patients exhibited disturbance in the oral phase (prolonged duration until swallowing) and oral residues, although disturbances in the pharyngeal phase and aspiration were mild.

According to a recent systematic review, no “high-quality” studies were identified for dysphagia in AD, compared with those of stroke, Parkinson disease, etc. [10], thus those discrepancies were needed to be clarified by further investigations.

In this study, we conducted a community-based investigation that aimed to clarify swallowing function in patients with MCI and those with dementia at various levels of severity. Major advantage points of the current study were:

1. A community-based design: the prevalence of dementia reported was 12.5% which coincided with the previous reports. The participants from this research field were not considered to be biased, since clinic-based MCI participants on other studies are easily closely biased to healthy adults or dementia people due to various methodology.
2. The Clinical Dementia Rating (CDR) assessment and neurological findings: board-certified neurologists (Masahiro Nakatsuka and Kenichi Meguro) and a CDR-Rater (Kenichi Meguro) assessed the CDR and neurological examinations [11,12].
3. Measures: tests were systematically administered to examine oral conditions, oral function, and subjective/objective swallowing function.

This is an observation study based on epidemiology, thus, no hypothesis has been considered.

## 2. Material and Methods

### 2.1. Participants

The protocol of selecting participants for this cross-sectional, observational study was as follows. The Kurihara Project was performed as a community-based stroke, dementia, and bed-confinement prevention for the old-old adults defined here as aged 75 years or older in Kurihara, an agricultural town in Miyagi Prefecture, northern Japan from 2008–2010. The total population in the city was about 76,708 and the population of the old-old was about 14,579 (17.9%; November 2010).

From among 255 communities in Kurihara, 19 were selected by the city officials, and they were asked to participate in the project (target population of 1254). These populations underwent (1) CDR assessment, (2) neuropsychological tests, (3) blood and urine tests, and (4) MRI scans.

Five hundred and ninety two people agreed to participate. The response rate was 47.0%. The reasons for refusal to participate were mainly “psychological” (30.1%) and “physical” (18.8%). Each age group achieved a statistically sufficient number for the confidence interval (C.I.) of 95%; considering that the prevalence of dementia was 10%. The people who cannot understand the verbal instruction were excluded.

Finally, 229 participants were analyzed for the eating and swallowing data completed, including 97 CDR 0 (healthy), 108 CDR 0.5 (MCI), and 24 CDR 1+ (dementia). We analyzed the relationships between the findings, eating, and swallowing, based on the database of the Kurihara Project performed from 2008 to 2010 [13].

The Kurihara Project was associated with GPs in the city. Suppose some participants were found to have some diseases, they were consulted by them. Demographics are described in Table 1.

### 2.2. CDR Assessments

For CDR assessment [11,12], a clinical team consisting of skilled physicians (neurologists and a psychiatrist) and skilled public health nurses determined the CDR for each participant and were blinded to the cognitive test results. They used a Japanese version of the questionnaire of the CDR scoring sheet. Before the physician interview, the public health nurses visited the subjects’ homes to evaluate their daily activities. Observations by family members regarding the subjects’ lives were described in a semi-structured questionnaire. Subjects who lived alone were visited frequently by public health nurses to evaluate their daily lives. The physicians interviewed the subjects to assess episodic memory, orientation, and judgment. Finally, with reference to the information provided by the family members, the CDR for each of the subjects was determined at a joint meeting of the physicians and public health nurses. One author (KM) was certified as a CDR rater at the Washington University at St Louis Alzheimer Disease Research Center Memory and Aging Project. Other physicians, psychologists and nurses were well educated for addressing and working with the participants.

### 2.3. Examinations

Tests were administered to examine oral conditions, oral function, and subjective/objective swallowing function.

#### 2.3.1. Oral Conditions

Tooth and artificial denture staining, tongue staining, and bad breath were evaluated by an examiner.

#### 2.3.2. Oral Function

Opening of the mouth, puffing out of the cheek, salivation, tongue protrusion range, tongue protrusion beyond the teeth, soft palate elevation, pharyngeal reflex, voice quality, presence of dysarthria, and oral diadochokinesis (OD) (Number of repeated pa-ta-ka per 10 s [14]) were evaluated by an examiner.

#### 2.3.3. Swallowing Function (Subjective)

We administered a questionnaire about swallowing (*Seirei* dysphagia screening questionnaire: [15]). This was designed so that the 15 questions would be answered by selecting 1 of 3 answers, as shown in Table 2.

#### 2.3.4. Swallowing Function (Objective)

As objective indexes of swallowing, we evaluated the following (see Table 3):

Repetitive Saliva Swallow Test (RSST [16,17]): Swallowing saliva for 30 s
Modified Water Swallow Test (MWST [18]): Drinking 3 mL of water
Food Test (FT [19]): Swallowing of 4 g jelly
  Swallowing time in MWST and FT

### 2.4. Ethics

Written informed consent was obtained from all of the participants with CDR 0 and 0.5 and from the family of those with CDR 0.5 and those with dementia. The ethical committee of Tohoku University Graduate School of Medicine approved the study (Ethical Approval Code #2007-414). An association with GPs were also considered in the project.

## 3. Results

Table 4 shows the results.

(1) Oral Condition

Tooth staining was increased with CDR 0.5 and 1.

(2) Oral Function

Pharyngeal reflex elimination and dysarthria were confirmed in many subjects with CDR 1.

(3) Swallowing Function (Subjective)
(4) Swallowing Function (Objective)

In FT, swallowing time was prolonged in the subjects with CDR 1, but no significant differences were confirmed among the 3 groups regarding choking and wet hoarseness in RSST, MWST, and FT.

Data from the individual CDR groups are shown below.

In the CDR 0.5 group, some decreases were confirmed regarding oral conditions, oral function, and swallowing function.

In the CDR 0.5 group, tooth staining, reduction in the OD (oral motion velocity), increase of points below the cut-off value in RSST and the questionnaire, and prolonged water swallowing time were confirmed.

In the CDR 1+ group, bad breath, pharyngeal reflex elimination, increase of disturbed soft palate elevation, and prolonged jelly swallowing time were confirmed.

In the CDR 0.5 and 1 group, no decrease was confirmed regarding the oral action range in the lips and tongue, MWST, and FT.

Table 5 summarizes the results. The arrows indicate the changes compared with the CDR 0 group.

The polypharmacy or antipsychotics use are considered to be related to swallowing disturbance. As Table 6 notes, no significant differences were observed between the CDR groups.

## 4. Discussion

### 4.1. Methodological Issues

Some methodological issues were considered. For the participant selection of the Kurihara Project [13], although the medical diagnosis was made and the number of participants was sufficient for a 95% confidence interval, the response rate was low. As some of refusals to participate were because of ‘psychological’ reasons, we felt that further enquiries of older adults might be culturally inappropriate. More than 50% of the refusals were for ‘physical’ reasons; most of these individuals already received Long-Term Care Insurance services, and they considered participating in the survey to be unnecessary. Despite these limitations, we believe that the results yielded information relevant to swallowing disorders of dementia.

### 4.2. Functions that Began to Deteriorate in the CDR 0.5 Group

It was suggested that OD and RSST would be related to executive function, which began to decrease in the CDR 0.5 group. Tooth staining might be affected by lowered instrumental activity of daily living (IADL) and oral residues. It was suggested that prolonged water swallowing time might be related to reduce function in the pharyngeal phase. We think it is important to promote oral care for older adults, even from the early stage as CDR 0.5. Observation should be initiated at CDR 0.5 with a focus placed on aspiration pneumonia.

### 4.3. Functions which Began to Deteriorate in the CDR 1+ Group

Bad breath might be affected by progressive IADL disturbance and oral residues. Pharyngeal reflex elimination and disturbed soft palate elevation might be related to a decrease in the phase close to the pharyngeal phase.

The prolonged jelly swallowing time was the same as the results of a preceding study. Prolonged swallowing time includes both delayed swallowing reflex and delayed swallowing action(s). Decreases in the pharyngeal phase might begin at CDR 0.5. These data were the same as the results in Horner’s report [9] on the characteristics of AD.

The current results were not changed after excluding the participants with possible sarcopenia based on the BML data (data not shown). We think that rehabilitation for dysphagia and prevention of aspiration pneumonia is important for older adults.

## 5. Conclusions

We report our findings of eating and swallowing-related functions in elderly local residents. We analyzed the relationships between the findings, eating, and swallowing, based on the database of the Kurihara Project. It was clarified that deterioration of swallowing function was confirmed, even in subjects with mild dementia, in addition to development of problems related to food intake. Older adults with dementia often develop aspiration pneumonia as a complication due to deterioration of swallowing function. It is important to prevent this condition by oral care activities in local communities, to maintain swallowing function.

## Figures and Tables

**Table 1:** Demographics.

	CDR 0	CDR 0.5	CDR 1+
*n* (male/female)	98 (40/58)	107 (47/60)	24 (9/15)
Age	78.8 (3.5)	80.8 (4.4) ^a^	82.6 (4.1) ^a^
Educational level	9.5 (2.1)	8.6 (1.7) ^a^	8.0 (1.2) ^a^
MMSE	25.5 (2.6)	22.9 (3.6) ^a^	16.0 (5.5) ^a,b^

Age, Education level, MMSE: mean scores (standard deviation). Statistical analysis: One-way ANOVA (post hoc tests; Bonferroni) in Age, Education level, MMSE. ^a^; significant difference with the CDR 0 group (*p* < 0.05). ^b^; significant difference with the CDR 0.5 group (*p* < 0.05). CDR = Clinical Dementia Rating. MMSE = Mini-Mental State Examination.

**Table 2:** Swallowing function (subjective).

	Questions			
(1)	Have you ever been diagnosed as having pneumonia?	A. Repeatedly	B. Once	C. No
(2)	Have you recently lost weight?	A. Clearly	B. Slightly	C. No
(3)	Do you feel difficulty in drinking?	A. Often	B. Sometimes	C. No
(4)	Do you choke on food?	A. Often	B. Sometimes	C. No
(5)	Do you choke on tea?	A. Often	B. Sometimes	C. No
(6)	Do you feel a sense of discomfort in your throat (feeling of having sputum in your throat) during/after eating, or in other circumstances?	A. Often	B. Sometimes	C. No
(7)	Do you feel some residual food in your throat?	A. Often	B. Sometimes	C. No
(8)	Do you take longer to eat than before?	A. Often	B. Sometimes	C. No
(9)	Do you have difficulty in eating something hard?	A. Often	B. Sometimes	C. No
(10)	Do you spill your food from the mouth?	A. Often	B. Sometimes	C. No
(11)	Do you have residual food in your mouth?	A. Often	B. Sometimes	C. No
(12)	Do you experience that food or acidic fluid comes back from the stomach to the throat?	A. Often	B. Sometimes	C. No
(13)	Do you feel that some food remains in your chest, or that you are choking with some food in your chest?	A. Often	B. Sometimes	C. No
(14)	Do you have experiences in which you could not go to sleep or you were awakened due to coughing during the night?	A. Often	B. Sometimes	C. No
(15)	Do you have a squeaky voice (hoarse voice, shattered voice, etc.)?	A. Always	B. Slightly	C. No

**Table 3:** Swallowing function (objective).

Repetitive Saliva Swallow Test (RSST)	Deterioration of Swallowing Function Was Confirmed When Swallowing ≤2 Times/30 s Was Confirmed.
Modified Water Swallow Test (MWST): Drinking of 3 mL water With no swallowing, choking and/or threatened breathing With swallowing, no choking and/or threatened breathing or wet hoarseness With swallowing, choking and wet hoarseness or no hoarseness With swallowing, no choking and no wet hoarseness In addition to 4, swallowing 2 times within 30 s after a cue is made	Possibility of aspiration was suggested when “1, 2 or 3” were confirmed.
Food Test (FT): Swallowing of 4-g jelly With no swallowing, choking and/or threatened breathing With swallowing, no choking and/or threatened breathing or wet hoarseness With swallowing, choking and favorable breathing and wet hoarseness or no hoarseness and/or moderate level of oral residues With swallowing, no choking and no wet hoarseness In addition to 4, swallowing 2 times within 30 s after a cue is made	Possibility of aspiration was suggested when “1, 2 or 3” was confirmed.

**Table 4:** Results.

Assessment	Swallowing	*n*	CDR 0	CDR 0.5	CDR 1+	χ^2^/F Value	*p* Value
MWST	Good (≥4) Poor (≤3)	227	97 1	98 7	23 1	-	0.103
FT	Good (≥4) Poor (≤3)	228	93 5	97 9	23 1	-	0.629
RSST	Good (≥3) Poor (≤2)	229	93 5	89 ^a^ 18	22 2	-	0.020
Pharyngeal reflex	Good Poor	228	63 35	65 42	21 ^a,b^ 2	-	0.013
Dysarthria	Good Poor	229	98 0	106 1	22 2	-	0.030
Tooth staining	Good Poor	227	83 14	75 ^a^ 32	10 ^a,b^ 13	χ^2^ = 18.7	<0.001
*Seirei*	Good Poor	229	90 8	83 ^a^ 24	18 6	χ^2^ = 8.9	0.012
Swallowing time (FT)	Mean SD	229	2.0 1.0	2.3 1.2	3.4 ^a,b^ 3.9	F = 7.1	0.001

MWST, FT, RSST, Pharyngeal reflex, and Dysarthria: Value are the number of subjects. Statistical analysis; Fisher’s Exact test, *p* < 0.05 (post hoc test, Fisher’s Exact test, *p* < 0.05/3). Tooth staining, and *Seirei*: Value are the number of subjects. Statistical analysis; Chi-square test, *p* < 0.05 (post hoc test, Chi-square test, *p* < 0.05/3). Swallowing time (FT): Value are the mean time and the standard deviation. Statistical analysis; One-way ANOVA, *p* < 0.05 (post hoc test, Bonferroni test, *p* < 0.05). ^a^; significant difference with the CDR 0 group (*p* < 0.05). ^b^; significant difference with the CDR 0.5 group (*p* < 0.05). CDR = Clinical Dementia Rating, MWST = Modified Water Swallow Test, FT = Food Test, RSST = Repetitive Saliva Swallow Test, SD = standard deviation; *Seirei*; Swallowing function (subjective) test (see Table 2).

**Table 5:** Summary of the results.

CDR 0	CDR 0.5	CDR 1+	One-Way ANOVA, Fisher’s Exact Test, *p* < 0.05
	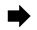	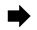	MWST, FT
	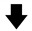	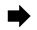	RSST, *Seirei*
	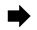	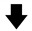	Pharyngeal reflex elimination and dysarthria, swallowing time (FT)
	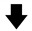	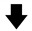	Tooth staining

Statistical analysis: CDR 0 vs. CDR 0.5 or CDR 1+; One-way ANOVA, *p* < 0.05 (post hoc test, Bonferroni test, *p* < 0.05); Chi-square test, *p* < 0.05 (post hoc test, Chi-square test, *p* < 0.05/3); CDR = Clinical Dementia Rating, MWST = Modified Water Swallow Test, FT = Food Test, RSST = Repetitive Saliva Swallow Test.

**Table 6:** Total number of medicine and psychotropic drugs.

		*n*	CDR 0	CDR 0.5	CDR 1+	χ^2^/F Value	*p* Value
Total number of medicine	Mean SD	216	5.1 3.7	5.6 4.1	6.2 3.0	0.9	0.429
Psychotropic drugs	No On medication	216	76 20	70 29	14 7	2.5	0.289

Total number of medicine: Value are the mean time and the standard deviation. Statistical analysis: One-way ANOVA, *p* < 0.05. Psychotropic drugs: Value are the number of subjects. Statistical analysis: Chi-square test, *p* < 0.05. CDR = Clinical Dementia Rating, SD = standard deviation.

## References

[1] Meguro K., Kasai M., Akanuma K., Meguro M., Ishii H., Yamaguchi S. (2014). Donepezil and life expectancy in Alzheimer’s disease: A retrospective analysis in the Tajiri Project. BMC Neurol..

[2] Chen H.J., Chen J.J., Chen C.Y., Lee M., Chang W.H., Huang T.T. (2019). Effect of an oral health programme on oral health, oral intake, and nutrition in patient with stroke and dysphagia in Taiwan: A randomized controlled trial. Int. J. Environ. Res. Public Health.

[3] Delwel S., Scherder E.J.A., Perez R.S.G.M., Hertogh C.M.P.M., Maier A.B., Lobbezoo F. (2018). Oral function of older people with mild cognitive impairment or dementia. J. Oral. Rehabil..

[4] Lin L.C., Wu S.C., Chen H.S., Wang T.G., Chen M.Y. (2002). Prevalence of impaired swallowing in institutionalized older people in Taiwan. J. Am. Geriatr. Soc..

[5] Clavé P., Rofes L., Carrión S., Ortega O., Cabré M., Serra-Prat M., Arreola V. (2012). Pathophysiology, relevance and natural history of oropharyngeal dysphagia among older people. Nestle Nutr. Inst. Workshop Ser..

[6] Yang E.J., Kim K.W., Lim J.Y., Paik N.J. (2014). Relationship between dysphagia and mild cognitive impairment in a community-based elderly cohort: The Korean Longitudinal Study on Health and Aging. J. Am. Geriatr. Soc..

[7] Ikeda M., Brown J., Holland A.J., Fukuhara R., Hodges J.R. (2002). Changes in appetite, food preference, and eating habits in frontotemporal dementia and Alzheimer’s disease. J. Neurol. Neurosurg. Psychiatr..

[8] Humbert I.A., McLaren D.G., Kosmatka K., Fitzgerald M., Johnson S., Porcaro E., Kays S., Umoh E.O., Robbins J. (2010). Early deficits in cortical control of swallowing in Alzheimer’s disease. J. Alzheimers Dis..

[9] Horner J., Alberts M.J., Dawson D.V., Cook G.M. (1994). Swallowing in Alzheimer’s disease. Alzheimer Dis. Assoc. Disord..

[10] Takizawa C., Gemmel E., Kenworthy J., Speyer R. (2016). A systematic review of the prevalence of oropharyngeal dysphagia in stroke, Parkinson’s disease, Alzheimer’s disease, head injury, and pneumonia. Dysphagia.

[11] Morris J.C. (1993). The Clinical Dementia Rating (CDR): Current version and scoring rules. Neurology.

[12] Meguro K., Ishii H., Yamaguchi S., Ishizaki J., Shimada M., Sato M., Hashimoto R., Shimada Y., Meguro M., Yamadori A. (2002). Prevalence of dementia and dementing diseases in Japan: The Tajiri project. Arch. Neurol..

[13] Meguro K., Tanaka N., Kasai M., Nakamura K., Ishikawa H., Nakatsuka M., Satoh M., Ouchi Y. (2012). Prevalence of dementia and dementing diseases in the old-old population in Japan: The Kurihara Project. Implications for Long-Term Care Insurance Data. Psychogeriatrics.

[14] Portnoy R.A., Aronson A.E. (1982). Diadochokinetic syllable rate and regularity in normal and in spastic and ataxic dysarthric subjects. J. Speech Hear. Disord..

[15] Ohkuma R., Fujishima I., Kojima C., Hojo K., Takehara I., Motohashi Y. (2002). Development of a questionnaire to screen dysphagia. Jpn. J. Dysphagia Rehab..

[16] Oguchi K., Saitoh E., Mizuno M., Baba M., Okui M., Suzuki M. (2000). The Repetitive Saliva Swallowing Test (RSST) as a screening test of functional dysphagia: Normal values of RSST. Jpn. J. Rehab. Med..

[17] Oguchi K., Saitoh E., Mizuno M., Baba M., Kusudo S., Tanaka T., Onogi K. (2000). The Repetitive Saliva Swallowing Test (RSST) as a screening test of functional dysphagia: Validity of RSST. Jpn. J. Rehab. Med..

[18] Tohara H., Saitoh E., Baba M., Onogi K., Uematsu H. (2002). Swallowing characteristics and tongue surface movements of persons with regard to pasty foods: A dysphagia evaluation system without videofluorographic study. Jpn. J. Dysphagia Rehab..

[19] Osawa A., Maeshima S., Tanahashi N. (2012). Food and liquid swallowing difficulty in stroke patients: A study based on the findings of food tests, a modified water swallowing test and videofluoroscopic examination of swallowing. Jpn. J. Rehab. Med..

